# Amelioration of cold-induced sweetening in potato by RNAi mediated silencing of *StUGPase* encoding UDP-glucose pyrophosphorylase

**DOI:** 10.3389/fpls.2023.1133029

**Published:** 2023-02-17

**Authors:** Sandeep Jaiswal, Krishnayan Paul, K. Venkat Raman, Saurabh Tyagi, Manjesh Saakre, Jyotsana Tilgam, Sougata Bhattacharjee, Joshitha Vijayan, Kalyan Kumar Mondal, Rohini Sreevathsa, Debasis Pattanayak

**Affiliations:** ^1^ ICAR-National Institute for Plant Biotechnology, New Delhi, India; ^2^ Post Graduate (PG) School, ICAR-Indian Agricultural Research Institute, New Delhi, India; ^3^ Division of Plant Pathology, ICAR-Indian Agricultural Research Institute, New Delhi, India

**Keywords:** cold induced sweetening, hpRNA, potato, reducing sugar, RNAi, UDP-glucose pyrophosphorylase

## Abstract

Cold-induced sweetening (CIS) is an unwanted physiological phenomenon in which reducing sugars (RS) get accumulated in potato (*Solanum tuberosum*) upon cold storage. High RS content makes potato commercially unsuitable for processing due to the unacceptable brown color in processed products like chips, fries, etc., and the production of a potential carcinogen, acrylamide. UDP-glucose pyrophosphorylase (UGPase) catalyzes the synthesis of UDP-glucose towards the synthesis of sucrose and is also involved in the regulation of CIS in potato. The objective of the present work was RNAi-mediated downregulation of the *StUGPase* expression level in potato for the development of CIS tolerant potato. Hairpin RNA (hpRNA) gene construct was developed by placing *UGPase* cDNA fragment in sense and antisense orientation intervened by GBSS intron. Internodal stem explants (*cv.* Kufri Chipsona-4) were transformed with hpRNA gene construct, and 22 transgenic lines were obtained by PCR screening of putative transformants. Four transgenic lines showed the highest level of RS content reduction following 30 days of cold storage, with reductions in sucrose and RS (glucose & fructose) levels of up to 46% and 57.5%, respectively. Cold stored transgenic potato of these four lines produced acceptable chip colour upon processing. The selected transgenic lines carried two to five copies of the transgene. Northern hybridization revealed an accumulation of siRNA with a concomitant decrease in the *StUGPase* transcript level in these selected transgenic lines. The present work demonstrates the efficacy of *StUGPase* silencing in controlling CIS in potato, and the strategy can be employed for the development of CIS tolerant potato varieties.

## 1 Introduction

Cold storage of potato (*Solanum tuberosum* L.) is an integral part of post-harvest handling of this semi-perishable crop. However, potato becomes sweet in taste due to the accumulation of reducing sugars (RS), a physiological process known as cold-induced sweetening (CIS) (Sowokinos, 1990). CIS occurs due to the cold-induced imbalance between the rate of breakdown of starch and its metabolism, resulting in higher glucose and fructose content in tubers (Sowokinos, 2001a; Blenkinsop et al., 2010). It is an undesirable physiological process as sweetened potato is not preferred for both table and processing purposes. Potato with a high amount of RS content develops brown to a black colour and bitter taste when fried at a higher temperature. Reducing sugars react with the amino group of free amino acids during frying or processing at high temperatures and develop dark pigmentation by a non-enzymatic reaction called Maillard reaction (Murata, 2021). Dark-coloured potato chips, fries, *etc*. taste bitter and are unsafe due to the presence of a high level of acrylamide, a byproduct of the Maillard reaction (Keijbets, 2008). Acrylamide has been classified as a ‘Probable carcinogen to humans (Group 2A) (Nursten, 2005; Borda and Alexe, 2011; Halford et al., 2011).

Due to a rapid expansion of the potato processing industry in India, the demand for quality raw materials throughout the year is also increasing and it is anticipated to rise from 6 million tonnes in 2025 to 25 million tonnes in 2050 (Singh, 2012). At present nearly 8.9% of the total potato produce is being processed, which is projected to be 10.76% by the year 2025 (CPRI Vision, 2050). However, the lack of supply of processing-grade potatoes throughout the year is still a major constraint for the potato processing industry (Das et al., 2021). Potato is kept in cold storage after harvest for yearlong supply and to avoid problems like price crashes and glut situations (Pradel et al., 2019). However, so far bred Indian potato cultivars including indigenous processing grade potato varieties by ICAR-CPRI, Shimla (i.e. ‘Kufri Chipsona1’, ‘Kufri Chipsona2’, ‘Kufri Chipsona3’, and ‘Kufri Chipsona4’) are not good cold chipper. Hence potato becomes unfit for processing after cold storage due to susceptibility to CIS and accumulation of a high amount of RS during cold storage (Pandey et al., 2009; Marwaha et al., 2010; Kumar, 2011; Raigond et al., 2018; Gupta et al., 2021). Therefore, the development of new cultivars with low RS build-up during cold storage or the improvement of existing genotypes with traits like cold/CIS tolerance is very important for the sustenance of potato processing industry. However, no resistant cultivar has indeed been developed so far by conventional breeding that could be regarded as good “cold chippers (Xiong et al., 2002; Hamernik et al., 2009). The biggest impediments towards the development of CIS resistant potato cultivars through conventional breeding are the lack of suitable germplasm, the genetic complexity of potato, heterozygosity, and polysomic tetraploid inheritance of this crop (Ortiz and Watanabe, 2004; Bradshaw et al., 2006; Hirsch et al., 2013). Therefore, genetic engineering is the most practical alternative for the amelioration of CIS in potato. Numerous efforts have been made to manipulate the expression of genes encoding the key regulatory enzymes, transcriptional factors, and other regulatory molecules that are directly or indirectly involved in CIS with variable degrees of success in reducing RS accumulation during CIS (Schwimmer et al., 1961; Sowokinos, 2001a; Geigenberger et al., 2004; Liu et al., 2010; Brummell et al., 2011; Liu et al., 2013; McKenzie et al., 2013; Zhang et al., 2013; Shivalingamurthy et al., 2018; Shi et al., 2021, Shi et al., 2022). Vacuolar acid invertase (vaINV), catalyzing the penultimate step, has been identified as one of the major factors involved in CIS (Zrenner et al., 1996; Matsuura-Endo et al., 2004; McKenzie et al., 2005; Liu et al., 2021). A few studies reported considerable improvement of CIS by down regulation of the *StvaINV* expression (Bhaskar et al., 2010; Wiberley-Bradford et al., 2014; Zhu et al., 2014; Hameed et al., 2018). However, it was observed that a high amount of sucrose gets accumulated in cold-stored *StvaINV* silenced potato, which imparted an undesirable sweet taste to chips (Pattanayak et al., 2009). This necessitates that an alternative strategy be devised to circumvent the problem of sucrose accumulation.

UGPase catalyzes the first reversible and committed step in sucrose synthesis through the formation of high-energy glycosyl nucleotide, UDP-glucose (Rees and Morrell, 1990; Spychalla et al., 1994; Borovkov et al., 1996). Double knockout mutant for UGPase alleles (*atugp1* and *atugp2*), confirmed the essential role of *AtUGPase* in carbohydrate metabolism in both the vegetative and reproductive phases in *Arabidopsis thaliana* (Park et al., 2010). During the initiation of CIS in potato tubers, the change in *StUGPase* expression and the concentration of UDP-Glc were found to be closely parallel to that of sucrose level, indicating the crucial role of UGPase in regulating carbon flux for sucrose biosynthesis in tubers during cold storage (Zrenner et al., 1993; Spychalla et al., 1994; Hill et al., 1996; Bagnaresi et al., 2008; Chen et al., 2008).To date, two *UGPase* alleles have been reported and designated as *UgpA* (A-II isozyme; UGP5) and *UgpB* (A-I isozyme; UGP3) in potato (Katsube et al., 1990; Spychalla et al., 1994; Sowokinos et al., 2018). Interestingly, the allelic ratios of *UgpA* and *UgpB* are reported to determine the degree of CIS resistance (Sowokinos, 2001b; Sowokinos et al., 2018). Despite a 96% reduction in UGPase activity through antisense inhibition in transgenic potato, no detrimental effect was observed in the growth and development of genetically engineered lines (Zrenner et al., 1993). While transgenic potato tubers expressing the *StUGPase* antisense construct were cold-stored, Spychalla et al. (1994) noticed a considerable shift in the sucrose concentration. Therefore, in the current study, we attempted RNAi-mediated downregulation of *StUGPase* to develop cold-chipping potato transgenic lines. Both sucrose and RS content reduced considerably in *UGPase* RNAi transgenic potato after cold storage at 4 °C for 30 days.

## 2 Materials and methods

Virus-free *in vitro* grown plantlet of potato cultivar Kufri Chipsona-4 (KC4), obtained from the Division of Crop Improvement, ICAR-Central Potato Research Institute, Shimla, was used as plant material for this study. The plantlets were cultured, multiplied, and maintained *in vitro* under the growth condition of 16h light-8h dark photoperiod cycle and 24 °C temperature. Internodal stem segments were used as explants for *Agrobacterium*-mediated transformation.

### 2.1 Development of *StUGPase*:RNAi gene construct

#### 2.1.1 Target selection

The *StUGPase* cDNA sequence of 1758 nt was retrieved from the NCBI (Sequence ID: D00667.1). The sequence was divided into four regions/fragments, and a similarity search was performed through the BLAST search in NCBI to identify the region with the least homology with other non-target genes. Moreover, the web tool dsCheck (http://dsCheck.RNAi.jp/) was used to identify the target region with maximum siRNA generation potential with the least off-targeting effect. The ~500 nt long sequences corresponding to the 450-949 nt region of the *StUGPase* cDNA were selected for the development of the hpRNA construct.

#### 2.1.2 Development of hpRNA *StUGPase* construct and binary vector cassette for potato transformation

**Figure 1:**
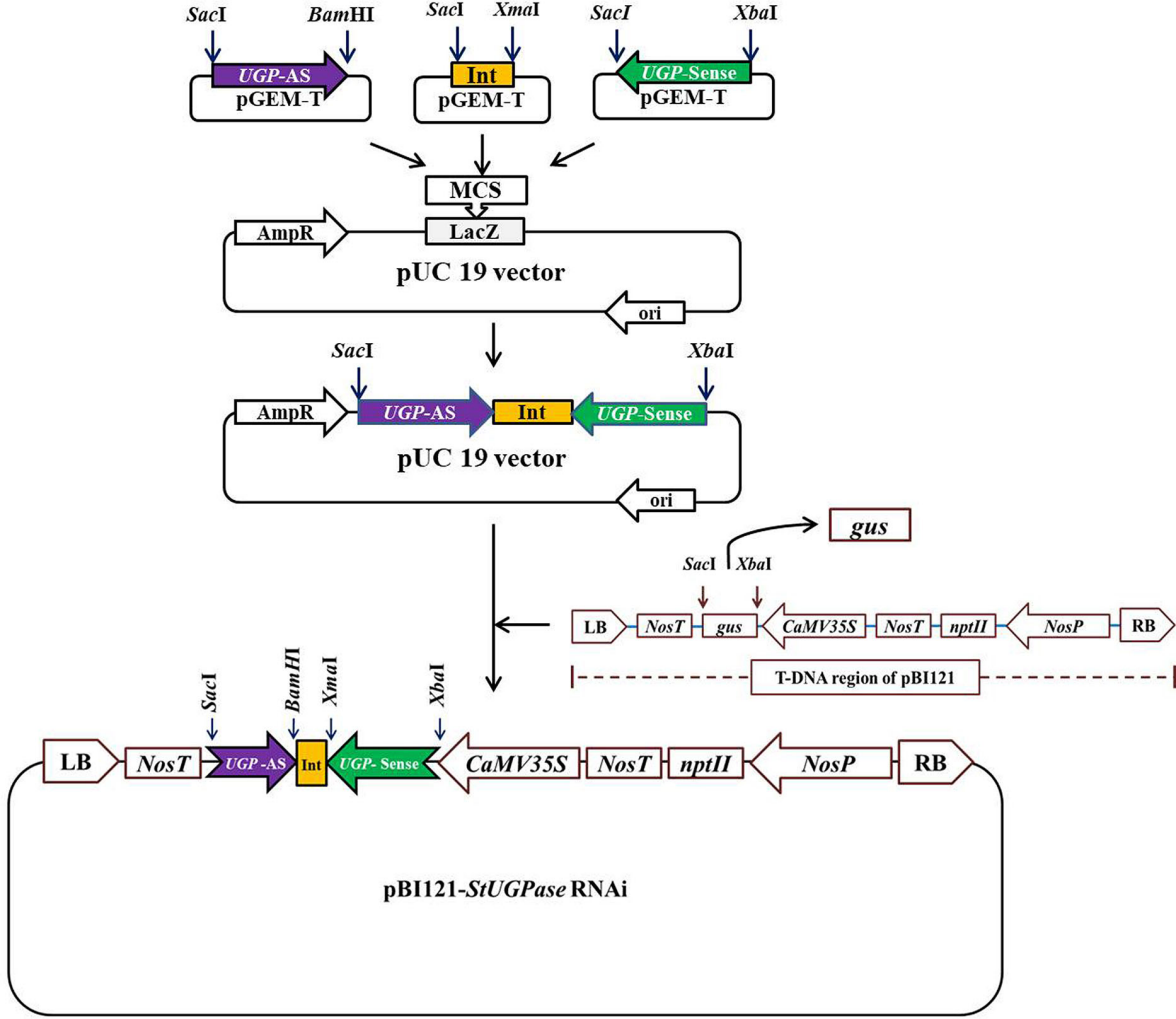
Outline of the strategy for the development of *StUGPase* hpRNA binary vector cassette, pBI121::*UGPase-*S:*GBSS-*Int:*UGPase*-AS (designated as pB::hpU) for potato transformation. The details of the cloning strategy have been described in Result section 3.1.

PureLink™ RNA Mini Kit (Invitrogen™) was used to extract total RNA from KC4 leaves following the manufacturer’s protocol. cDNA was synthesized from 1 µg of total RNA using PrimeScript™ 1st strand cDNA Synthesis Kit (Takara Bio ink). DNA fragments of ~500 bp were amplified from KC4 cDNA using specially designed *StUGPase* sense and antisense specific primers having specific restriction sites incorporated in them (**Supplementary Table 1**) using a thermal cycler (Eppendorf, Germany). The amplified fragments were ligated, individually onto the pGEM T-Easy vector. Potato Granule Bound Starch Synthase (GBSS) intron of 105 nt corresponding to 3821-3925 nt of GBSS gene sequence (*StGBSS*; NCBI Accession ID: X58453) was amplified with specific sets of primers (**Supplementary Table 2**) containing suitable restriction enzyme sites and ligated onto the pGEM T-Easy vector. After sequence confirmation the *UGPase* sense (UGPase-S) fragment was taken out from the pGEM T- Easy vector by *Xba*I-*Sac*I digestion and ligated onto *Xba*I-*Sac*I digested pUC19 vector to get pUC19::*UGPase*-S. Similarly, 105 bp potato *GBSS* intron was taken out by *Xma*I-*Sac*I digestion of pGEM T-Easy vector and cloned onto the pUC19::UGPase-S, linearized by *Xma*I-*Sac*I digestion, to get pUC19::UGPase-S:*GBSS*-Int. UGPase antisense (UGPase-AS) fragment was finally subcloned onto *Bam*HI*-Sac*I sites of the pUC19::*UGPase*-S:*GBSS*-Int to get pUC19::*UGPase-*S:*GBSS*Int:*UGPase*-AS (designated as pU::hpU; hereby the hpRNA *StUGPase* construct is abbreviated as hpU). Each step of the assembly was confirmed by restriction digestion and sequencing. Finally, the hpU construct was subcloned onto pBI121 binary vector linearized by *Xba*I- *Sac*I digestion to get the binary vector cassette pBI121::hp*UGPase* (hereby, designated as pB:hpU; **Figure 1**). The constructed recombinant binary vector cassette was confirmed by restriction analysis and sequencing. The binary vector was then transformed into *A. tumefaciens* strain EHA105 by freeze-thaw method (Höfgen and Willmitzer, 1988). Colonies harboring pB:hpU were confirmed by PCR and then used for potato transformation.

### 2.2 Potato transformation

*in vitro* grown potato plantlets (20-23 days old) were used as a source of internodal stem explants. About 100 such explants were placed on Petri plates containing filter paper (90 mm Whatman No.3) placed on pre-culture medium (M.S. medium without any hormone or antibiotics, pH- 5.8) for two days in dark conditions. The pre-cultured internodal stem explants were taken out from Petri plates and dipped in diluted (1:5) *Agrobacterium* culture, harbouring pB:hpU binary vector cassette, for 1 min in an aseptic Petri plate. The plate containing explant with *Agrobacterium* culture was shaken slowly in rotational movement. The explants were dried on sterile filter papers and kept on the same pre-culture plates for two days for co-cultivation in the dark. After that, transformed internodal explants were sub-cultured on selection medium [M.S. basal medium (PT021, Himedia) supplemented with 2 mg/l Ca-pantothenate, 3 mg/l GA_3_, 3 mg/l *Trans*-Zeatin Riboside, 0.05 mg/l IAA, 50 mg/l kanamycin, 250 mg/l cefotaxime and 250 mg/l carbenicillin, 20g/l sucrose, pH = 5.8], and the plates were kept at 24 °C under 16 hours light and 8 hours darkness. After 6-8 days, the cultures were shifted into fresh regeneration plates to avoid agro-contamination followed by regular sub-culturing after 10-15 days. After 30-40 days of selection, a tiny adventitious shoot/plantlet proliferating from one/both ends of the explant can be visible. The shoots were allowed to grow up to 2-3 cm and then excised and transferred into tubes containing selective propagation medium for a better shoot and root growth and kept at 24 °C. After rooting, the plants are allowed to grow for 3-4 weeks and multiplied at regular intervals.

### 2.3 Screening and selection of putative transgenic lines

The putative transformants were screened by PCR for transgene integration using specific sets of primers (UGPase-S F & GBSS-Int R; **Supplementary Table 2**) to amplify 600 bp fragment encompassing *UGPase*-S and *GBSS*-Int. PCR amplification (**Supplementary Table 3**) was carried out in a thermal cycler (Eppendorf, Germany) programmed with a hot start at 94°C for 5 min, followed by 35 cycles of 94°C for 1 min, 55°C for 1 min and 72°C for 1.5 min and a final extension at 72°C for 7 min. The amplified PCR products were resolved on 1% agarose gel, visualized on a UV- trans-illuminator, and photographed using a gel documentation system. Transgene expression in the PCR-confirmed transgenic lines was confirmed by RT-PCR. Total RNA was extracted from the *UGPase* RNAi potato lines and wild-type control potato plants using the Spectrum^TM^ Plant Total RNA Extraction kit (Sigma, USA) according to the manufacturer’s protocol. The cDNA was synthesized from 800 ng of total RNA using the PrimeScript^TM^ single-strand cDNA Synthesis Kit (Takara Bio Ink). RT-PCR was done using the same primer set used for PCR analysis. The amplified RT-PCR products were electrophoresed on 1% agarose gel, visualized on U.V.- transilluminator, and photographed using a gel documentation system.

### 2.4 Net house trial of *in-vitro* grown *UGPase*-RNAi transgenic lines

The RT-PCR positive putative transgenic lines were further multiplied *in-vitro* and were grown in soil in a transgenic net house under short-day winter conditions. Haulm cutting was done 120 days after planting (DAP), and tubers were collected after 7 days of haulm cutting. The baby tubers so obtained were kept in a cold chamber in March, and taken for sprouting in the first week of October. Sprouted tubers were planted in soil in a transgenic net house in the last week of October under the natural short-day winter condition of Delhi, India. Haulm cutting was done 120 days after planting and harvesting was done 7 days after haulm cutting. Processing attributes were analyzed at fresh harvest, and the rest of the tubers were stored at 4°C in a controlled environment growth chamber (Percival- Scientific, USA). Uniform-size tubers were taken out from the cold chamber after 30 days of cold storage for analysis of cold chipping attributes.

### 2.5 Evaluation of cold chipping attributes of potato pre and post-cold-storage

The chipping attributes of the control, and transgenic lines, were assessed at various stages like fresh harvest (without cold treatment), 30 days of cold storage at 4°C, and 21 days of reconditioning at room temperature. Six freshly harvested tubers from each transgenic line were randomly selected for the preparation of chips. Potato chips prepared by deep-frying at 184 °C in groundnut oil were assigned a colour score on a scale of 1-10 based on visual inspection as per the colour score reference chart for potato chips developed by Ezekiel et al. (2003). Chips with a colour score of 1 are the lightest in colour and chips with a score of 10 are the darkest in colour. Chips with colour scores of 1-3 are preferred and are considered acceptable as per the standard norm of the processing industry.

### 2.6 Sugar estimation

Total sugar was extracted following the protocol of Viola and Davies (1992) with slight modifications. Tuber samples were ground to a fine powder in a pre-chilled pestle and mortar, and about 200 mg of the sample was taken in 2 ml Eppendorf tubes containing 1 ml of 80% ethanol. Sampling was done in replication of three for each transgenic line. Tubes were kept at 4°C overnight with intermittent vortexing. The next morning the mixture was boiled at 80°C for 10 minutes and then allowed to cool at RT. Tubes were centrifuged for a brief period at 3000xg for 2 min, and the clarified supernatant was transferred to fresh tubes. The above step of ethanol extraction was repeated twice with the same sample. The collected clarified supernatant was kept in a vacuum evaporator (CentriVap Centrifugal Concentrator) at 40° C till the entire solution is evaporated. Sugar was dissolved in 200 ul of water and the soluble sugar (glucose, fructose, and sucrose) content was estimated using the Sucrose/D-Fructose/D-Glucose Assay Kit (Product code: K-SUFRG from Megazyme). All the reagents were prepared as per the manufacturer’s instructions (https://www.megazyme.com/documents/Assay_Protocol/K-SUFRG_DATA.pdf) and were stored at the appropriate temperature. The 1/10 diluted sugar sample was used for the assay reaction in triplicate and the absorbance reading during enzymatic assay was taken in a spectrophotometer at 340 nm.

### 2.7 Molecular characterization of the selected transgenic potato lines

#### 2.7.1 Southern hybridization analysis

Southern hybridization was done to confirm the transgene integration and the transgene copy number in the genome of *UGPase* RNAi transgenic potato lines. Approximately 15 µg of genomic DNA per sample was completely digested with 5 µl of *Eco*RI (*Eco*RI-HF^®^ 20U/ µl) in a 50 µl reaction and resolved on 0.8 % Agarose gel (without ethidium bromide) in 1X TAE buffer. Southern blot preparation was done according to Russell and Sambrook (2001) using a positively charged nylon membrane (Hybond N^+^, Amersham, U.K.). The DNA blotting (capillary transfer) was carried out at room temperature for 16 hr. The membrane was carefully removed from the gel, rinsed in 2X SSC (0.3 M NaCl, 0.03 M Trisodium Citrate, pH 7.0), and placed on Whatman 3 paper for air-drying. The membrane was fixed at 22 J/cm for 2 min using Stratalinker UV-crosslinker (Stratagene, U.K.). A 790 bp of DIG-labelled probe was PCR amplified by using *nptII* gene-specific primers. Probe preparation, prehybridization, hybridization, and detection were performed by following the protocol provided by DIG Luminescent Detection Kit (Roche). The hybridized membrane was exposed to X-ray film (Fujifilm, Kodak) in an intensifying cassette under dark conditions. The cassette was placed for 2 hours, and the exposed X-ray film was developed to visualize the results.

#### 2.7.2 Northern hybridization analysis

RNA was isolated from the cold stored tubers following the protocol developed by Kumar et al. (2007). For small RNA northern blot analysis, a 5% metaphor gel was prepared and 90 µg total RNA per sample was loaded for separation. RNA samples were separated at 70-75 V at 4°C for 3-4 hr and then transferred to a positively charged nylon membrane (N^+^, Bright Star-Plus, Applied Biosystem) through capillary blotting setup, same as described for Southern blot transfer in the section 2.7.1. The capillary transfer of RNA was carried out for 7-8 hours while maintaining the transfer buffer temperature at 4˚C. For cross-linking, EDC reagent was used as described by Pall and Hamilton (2008) in place of U.V.cross-linking. A PCR DIG-Probe Synthesis Kit with DIG-dUTP from Roche was used for the PCR labelling of a 500 nt long probe specific to the target sequence using *StUGPase-*S-specific primers by following the manufacturer’s instructions. Pre-hybridization, hybridization, and band detection were performed following the protocol provided by DIG Luminescent Detection Kit (Roche, Basel, Switzerland).

#### 2.7.3 Quantitative real time-PCR

For detecting the downregulation of *StUGPase* expression in transgenic lines compared to the control at various treatment conditions (fresh vs. cold treatment), qRT-PCR analysis was performed using *StUGPase*-specific primers. For tuber tissue, sampling was done at fresh harvest as well as after 30 days of cold storage. qRT-PCR reactions were performed in the Agilent qPCR system under the following conditions, initial denaturation at 95°C for 5 minutes, followed by 40 cycles of 95˚C for 30 sec, 62˚C for 30 sec and 72˚C for 20 seconds. The reactions were carried out using gene-specific real-time primers (**Supplementary Table 4**) and the *StEF*1α gene as an internal control. The relative expression levels were calculated using the 2^−ΔCt^ method (Pfaffl, 2001). The specificity of the PCR amplification was checked through melt curve analysis.

## 3 Results

### 3.1 Development of hpRNA *StUGPase* gene construct and binary vector cassette(s) for potato transformation

The strategy adopted was to develop the *StUGPase* hpRNA gene construct by cloning the target sequence in an inverted repeat (IR) orientation intervened by potato “granule-bound starch synthase (GBSS)” intron. The complete cDNA sequence of *StUGPase* (Sequence ID: D00667.1) was retrieved from GenBank (**Supplementary Figure 1**). For the development of hpRNA construct, a 500 nt sequence corresponding to the 450-949 region of *StUGPase* cDNA (**Supplementary Figure 2**) was selected using the web tool dsCheck (http://dsCheck.RNAi.jp/). Potato genomic DNA sequence of 105 nt corresponding to 3821-3925 nt of potato GBSS genomic DNA sequence (Sequence ID: X58453) comprised of 13 nt GBSS exon-13 (5′ exons), 85 nt GBSS intron-13 (last intron), and 6 nt GBSS exon 14 (3′ exons) was used as an intervening sequence for inverted repeat (IR) gene construct (**Supplementary Figure 3**).

The targeted region of *StUGPase* was RT-PCR amplified using two sets of primer pairs (**Supplementary Table 1**), and the 500 bp long amplified DNA fragments, designated as sense (S) and antisense (AS), were ligated separately onto pGEM T-Easy vector through TA-cloning. Similarly, the 105 bp potato GBSS intron was PCR amplified from KC-4 genomic DNA and cloned onto the pGEM T-Easy vector. The *StUGPase*-S, *StGBSS*-Int, and *StUGPase*-AS fragments were confirmed by sequencing, and then sequentially subcloned onto the pUC19 vector to generate *UGPase* hpRNA construct (**Figure 1**). At every step of assembly, the clones (pUC19::*StUGPase-S*, pUC19::*StUGPase*-S:*StGBSS-Int*, and pU::hpU) were confirmed by restriction digestion and sequencing (**Figure 1**). The assembled ~1100 bp long hpU fragment was released from the pU::hpU by *Xba*I-*Sac*I digestion and then subcloned onto the pBI121 backbone, generated by replacing the GUS coding region by *Xba*I-*Sac*I digestion to get pB::hpU RNAi binary vector cassette. Development of pB::hpU was confirmed through different combinations of restriction digestion (data not shown) and then introduced into *Agrobacterium tumefaciens* EHA105 competent cells for potato transformation.

### 3.2 Potato transformation

**Figure 2:**
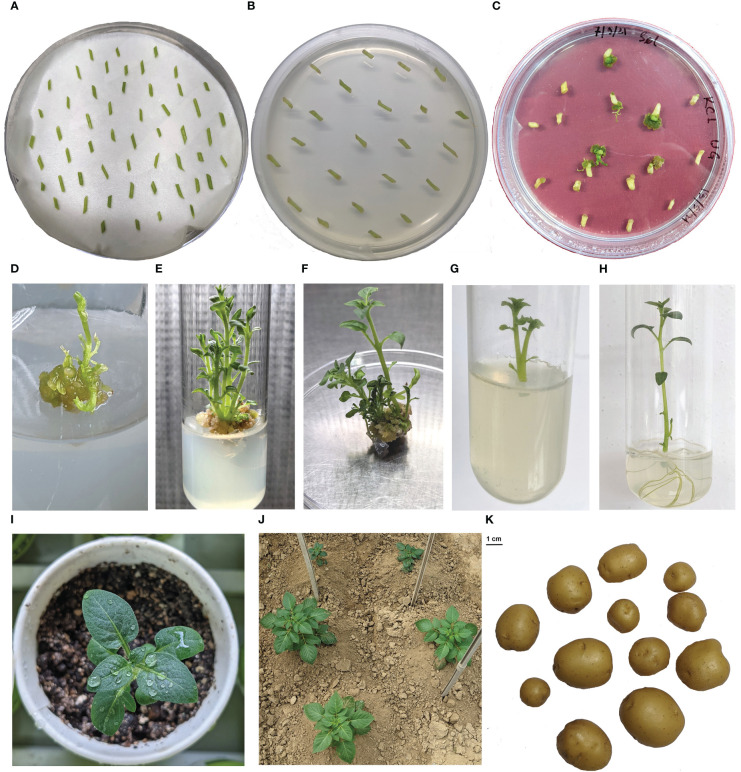
Transformation, regeneration, and net house trial of *UGPase* RNAi potato transgenic lines of KC4. **(A)** pre-culture, **(B)** selection, **(C)** regeneration, **(D-F)**. shoot elongation, **(G)** transfer into rooting media, **(H)** rooting, **(I)** hardening, **(J)** net house grown plants, **(K)** harvested tuber.

About 800 inter-nodal stem explants of the potato cultivar, KC4, were used for the *Agrobacterium*-mediated genetic transformation technique and 27 putative transformants were developed. These putative transformants were screened by PCR and RT-PCR and 22 *UGPase* RNAi lines were obtained (**Figure 2**). A DNA fragment of ~600 bp corresponding to the cloned sequence of *StUGPase*-S and *StGBSS*-Int region was amplified from the 22 *UGPase* RNAi transgenic lines but not from the non-transgenic control (**Supplementary Figures 4**, **5**). These RNAi transgenic lines were grown in the transgenic net house as short-day winter crop. During the net house trial, normal growth and development were observed in all the *UGPase* RNAi transgenic lines, and no morphological abnormalities were seen in any of the transgenic lines (**Supplementary Figure 6**).

### 3.3 Net house trial

*In vitro* multiplied plantlets of the 22 *UGPase* RNAi transgenic lines and the non-transgenic KC4 control (approx. 20 plants for each line) were grown in the net house as a short-day winter crop. Tubers were harvested in February after 120 days of planting and kept in cold storage for seed purpose as they were small and of varying size. The tubers were taken from cold storage in the first week of October and kept at room temperature for sprouting. Sprouted seed tubers were planted in the last week of October in soil in the net house under natural short-day conditions. Haulm cutting was done after 120 days of planting, and harvesting was done 7 days after haulm cutting. The yield attribute of the RNAi transgenic lines was found variable across the lines and significantly different when compared to non-transgenic control. Out of the total of 22 lines, the highest tuber yield was recorded in UG4 (327.04 g/plant), whereas the lowest was recorded in UG11 (91.60 g/plant) (**Supplementary Table 5**). After the evaluation of cold chipping abilities, a total of four transgenic lines (UG14, UG18, UG19, and UG21) were selected for further analysis. The average tuber yield of the four selected lines (272.60 gm/plant) was found similar to the average yield of the control line (305.06 gm/plant).

### 3.4 Evaluation of processing attributes

#### 3.4.1 Chip color

**Figure 3:**
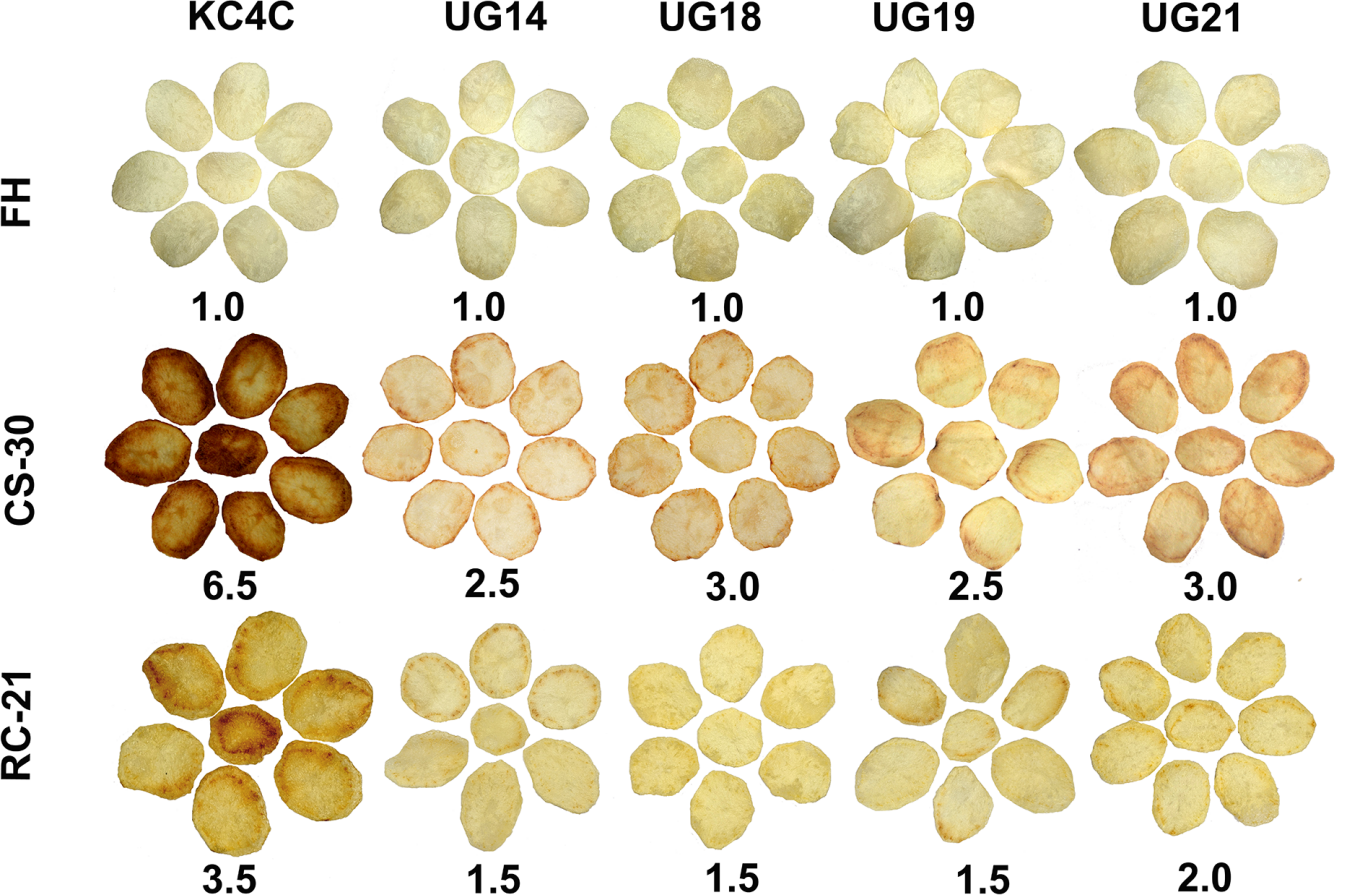
Cold chipping performance of the four best performing *UGPase* RNAi potato transgenic lines of KC4 at fresh harvest (FH), after 30 days of cold storage (CS) and after 21 days of room temperature reconditioning (RC) of the cold stored tubers. The number below chips of each line indicates chip colour score.

Chips were prepared from both fresh harvest and cold stored tubers. At fresh harvest, all the RNAi transgenic lines and non-transgenic control produced desirable light colour chips with a score of 1.0. However, chips prepared after 30 days of cold storage displayed drastic differences in chip colour scores ranging from 2.5 to 6.5. KC4 control tubers produced the darkest color chips (colour score 6.5) after 30 days of cold storage. In general, there was deterioration in chip colour in all the *UGPase* RNAi lines after cold storage compared to that of fresh harvest. The cold-chipping performance showed that out of 22 RNAi transgenic lines evaluated, chips produced from the four RNAi lines, UG14, UG18, UG19, and UG21, had acceptable light colour after 30 days of cold storage. The chip colour score for the two *UGPase* RNAi lines, UG14 and UG19, was 2.5, while for the other two RNAi lines, UG 18 and UG 21, it was 3.0 (**Figure 3**; **Supplementary Table 6**). There was considerable improvement in chip colour in all the RNAi lines after reconditioning of cold-stored tubers for 21 days at room temperature. Chips colour for the three best performing RNAi lines, UG14, UG18, and UG19 was 1.5, while for the other best performing RNAi line, UG21, it was 2.0. In contrast, the non-transgenic counterpart produced chips with colour score of 3.5 after reconditioning (**Figure 3**).

#### 3.4.2 Soluble sugar content

**Figure 4:**
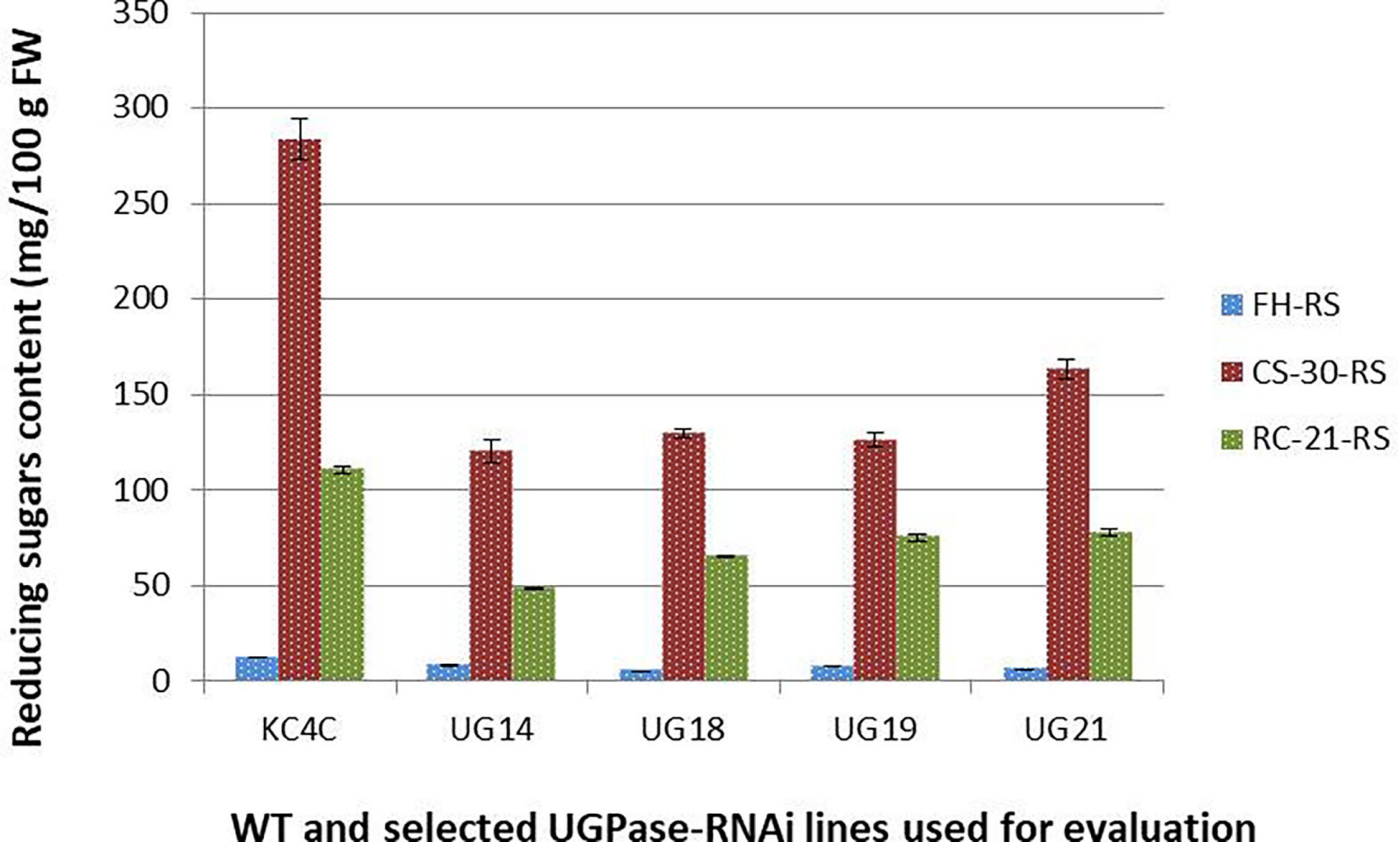
Reducing sugar content in the *UGPase* RNAi potato transgenic lines of KC4. KC4C, Kufri Chipsona-4 control; UG14, UG18, UG19, and UG21 are the four selected *UGPase* RNAi transgenic lines of KC4.FH, Fresh harvest; CS-30, cold stored for 30 days; RC-21, 21 days reconditioning at room temp after cold storage.

Total soluble sugars [Reducing sugar (RS), glucose and fructose; and sucrose] content of the four best performing *UGPase* RNAi lines were estimated from tubers at fresh harvest, after cold storage, and after reconditioning. In general, RS and sucrose content was much less in all the four selected RNAi transgenic lines at all three treatment conditions studied. At harvest, RS content in the four selected RNAi lines ranged from 4.9-8.13 mg/100g fresh weight compared to 12mg/100g fresh weight in KC4 non-transformed control. The maximum reduction in RS content was observed in UG18 (59.31%) followed by UG21 (50.12%) and UG19 (37.58%), whereas the least reduction was recorded in UG14 (32.41%) (**Figure 4**; summarised in the **Supplementary Table 6**). Around 15-25 times increase in RS level was observed in both the RNAi transgenic and control tubers after cold storage for 30 days. However, the extent of increase in RS content was 54.09% to 57.52% lesser in the four RNAi transgenic lines than that of the control after cold storage. The RS content in cold-stored tubers of these four *UGPase* RNAi lines ranged from 120 mg to 163 mg per 100 g FW, whereas it was more than 250 mg per 100 g FW in the control tubers (**Figure 4**). RS level reduced considerably in both the RNAi lines and control after reconditioning. RS level varied from 48.25 mg to 77.7 mg per 100 g FW in the RNAi lines, whereas it was 110.6 mg per 100 g FW in the control line after reconditioning (**Figure 4**). The reduction in RS content in the RNAi lines was 37.4% to 59.3% over that of 30-day cold storage (**Figure 4**).

**Figure 5:**
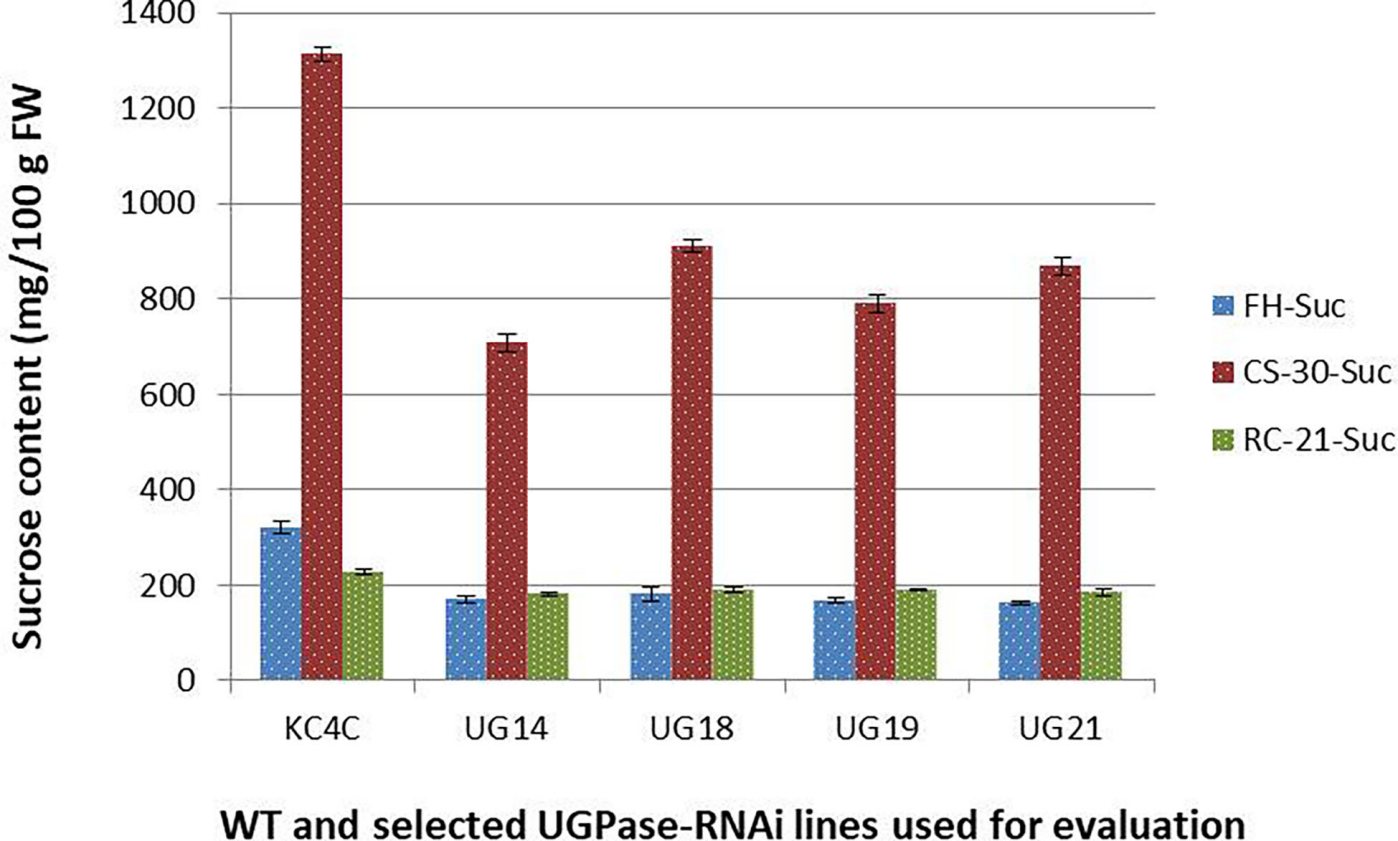
Sucrose content in the *UGPase* RNAi potato transgenic lines of KC4. KC4C, Kufri Chipsona-4 control; UG14, UG18, UG19, and UG21 are the four selected *UGPase* RNAi transgenic lines of KC4. FH, Fresh harvest; CS-30, cold stored for 30 days; RC-21, 21 days reconditioning at room temp after cold storage.

Similarly, there was a significant reduction in sucrose level in the *UGPase* RNAi transgenic lines than that of the non-transformed control. At harvest, the control tuber had sucrose content of 320.16 mg/100 g fresh weight, and the average sucrose content of the RNAi lines was 170.23 mg/100 g fresh weight which is around 43% less than that of the control (**Figure 5**). The highest reduction of sucrose content was observed in line UG21 (50%) followed by UG19 (47.55%), UG14 (46.77%), and UG18 (43.55%). After cold storage at 4° C for 30 days, sucrose content increased upto 910 mg/100g FW in the *UGPase* RNAi transgenic lines, while it was 1314 mg/100g FW in the control tubers implying a 4-5.4 times increase in sucrose content under cold stored condition than that of fresh harvest (**Figure 5**). The sucrose level in the RNAi transgenic lines was 31-46% lesser than that of the non-transgenic counterpart. The highest reduction was noticed in line UG14 (46.12%), followed by UG19 (39.9%), UG21 (33.9%), and UG18 (30.7%). When the sucrose content of tubers after reconditioning was compared with that of fresh harvest, the reconditioned tubers were found to possess slightly higher sucrose levels than that of the control tuber. Following the trend, the sucrose content of the transgenic lines was 17-22% lower than that of the non-transgenic counterpart after reconditioning (**Figure 5**).

### 3.5 Molecular characterization of selected transgenics

#### 3.5.1 Southern analysis

**Figure 6:**
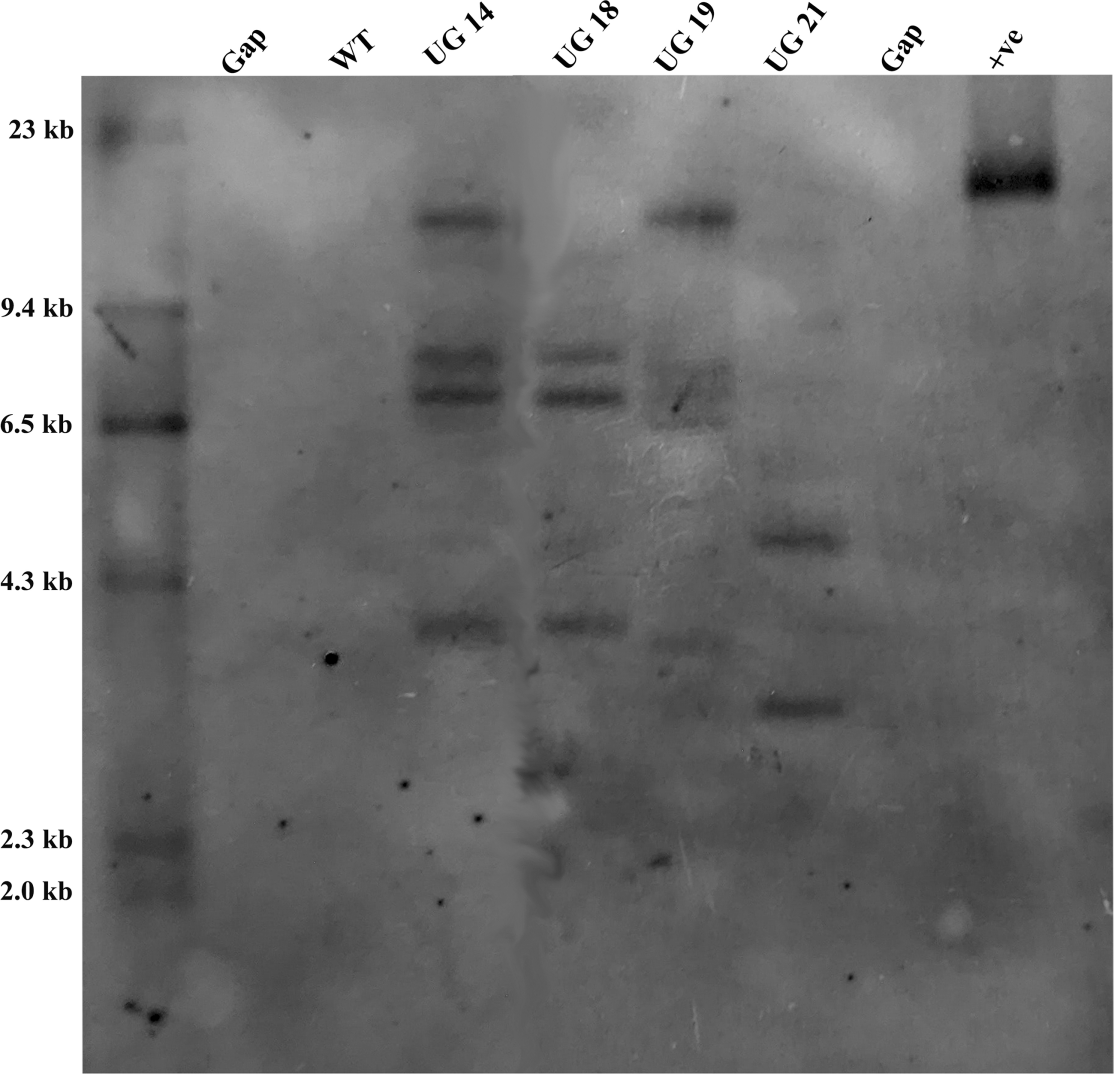
Southern blot analysis of the *UGPase* RNAi transgenic potato lines of KC4. Lanes: WT, wild type control; UG14, UG18, UG19, UG21 represent transgenic potato lines; +ve, positive control.

The four *UGPase* RNAi potato lines were analyzed for confirmation of gene integration and copy number by Southern hybridization. Genomic DNA isolated from leaves of the four RNAi transgenic lines and non-transformed KC4 control was digested with *Eco*RI, which had a single recognition site within the T-DNA region of pB:hpU binary vector. PCR amplified 790 bp *npt*II fragment was labeled with DIG-dUTP and used as a probe. The selected four *UGPase* RNAi transgenic potato lines carried 2-5 copies of the transgene in their genome. Out of four selected transgenic potato lines, UG19 had five, UG14 had four, UG18 had three and UG21 had 2 copies of the transgene (**Figure 6**).

#### 3.5.2 Small RNA Northern blot analysis

**Figure 7:**
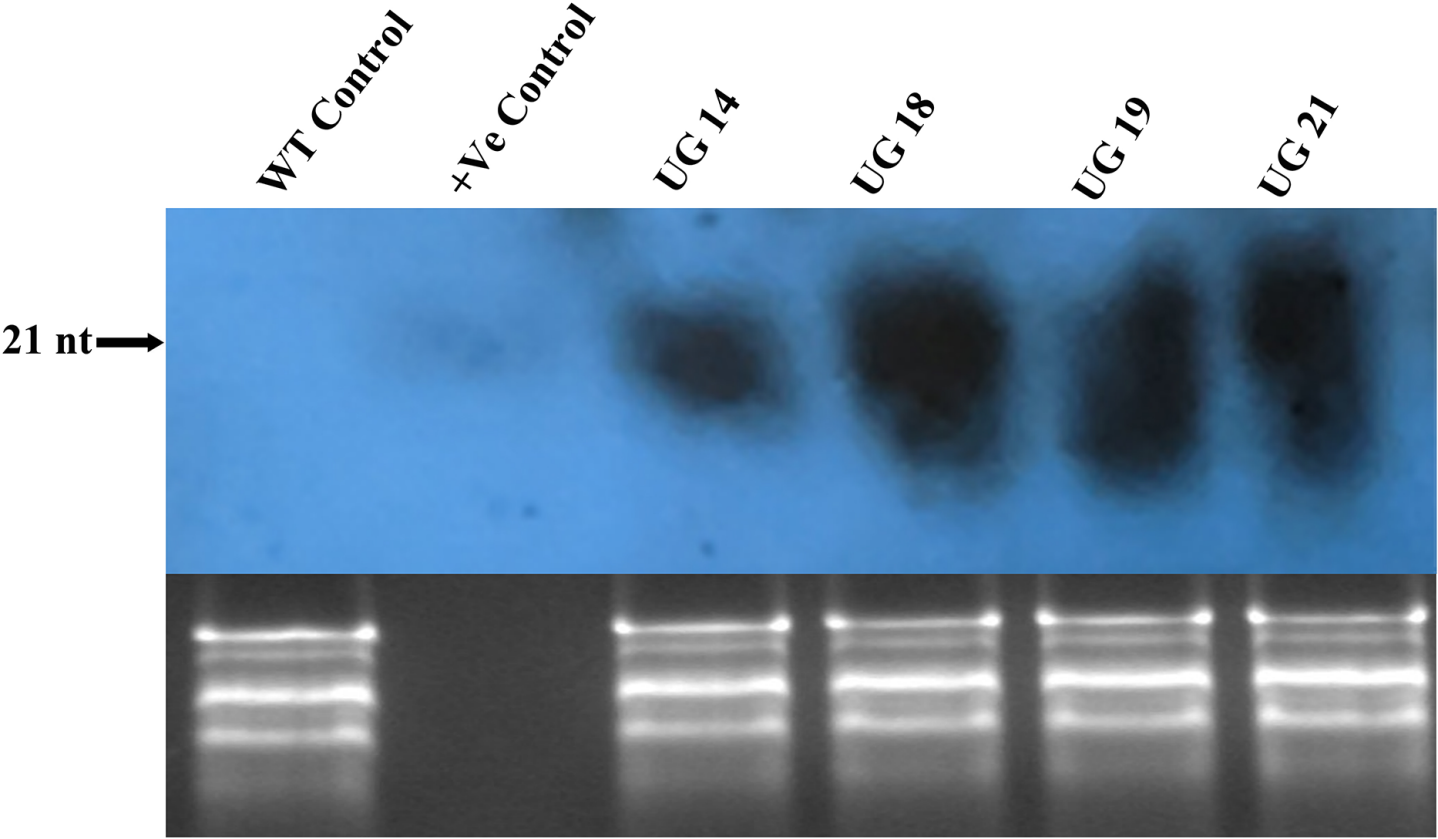
Small RNA northern blot of the *UGPase* RNAi transgenic potato lines of KC4. siRNA band corresponding to ~21nt long positive control oligo was detected in the fourRNAi transgenic lines. Lanes: Lanes: WT, wild type control; +ve, positive control; UG14, UG18, UG19, UG21 represent transgenic potato lines.

Small RNA northern hybridization was done to detect the accumulation of siRNA in the RNAi transgenic lines. Total RNA isolated from the cold stored tubers was resolved on metaphor agarose gel and blotted onto the membrane, which was probed with randomly labeled *UGPase-S* cDNA. Positive control of 21nt long oligo with sequence similarity with UGPase mRNA target region was used as control. A prominent band of ~21 nt long was detected in all the four RNAi transgenic lines indicating *UGPase*-specific siRNA accumulation (**Figure 7**). No *UGPase*-specific siRNA band was observed in case of the non-transgenic control.

#### 3.5.3 Real-time PCR

**Figure 8:**
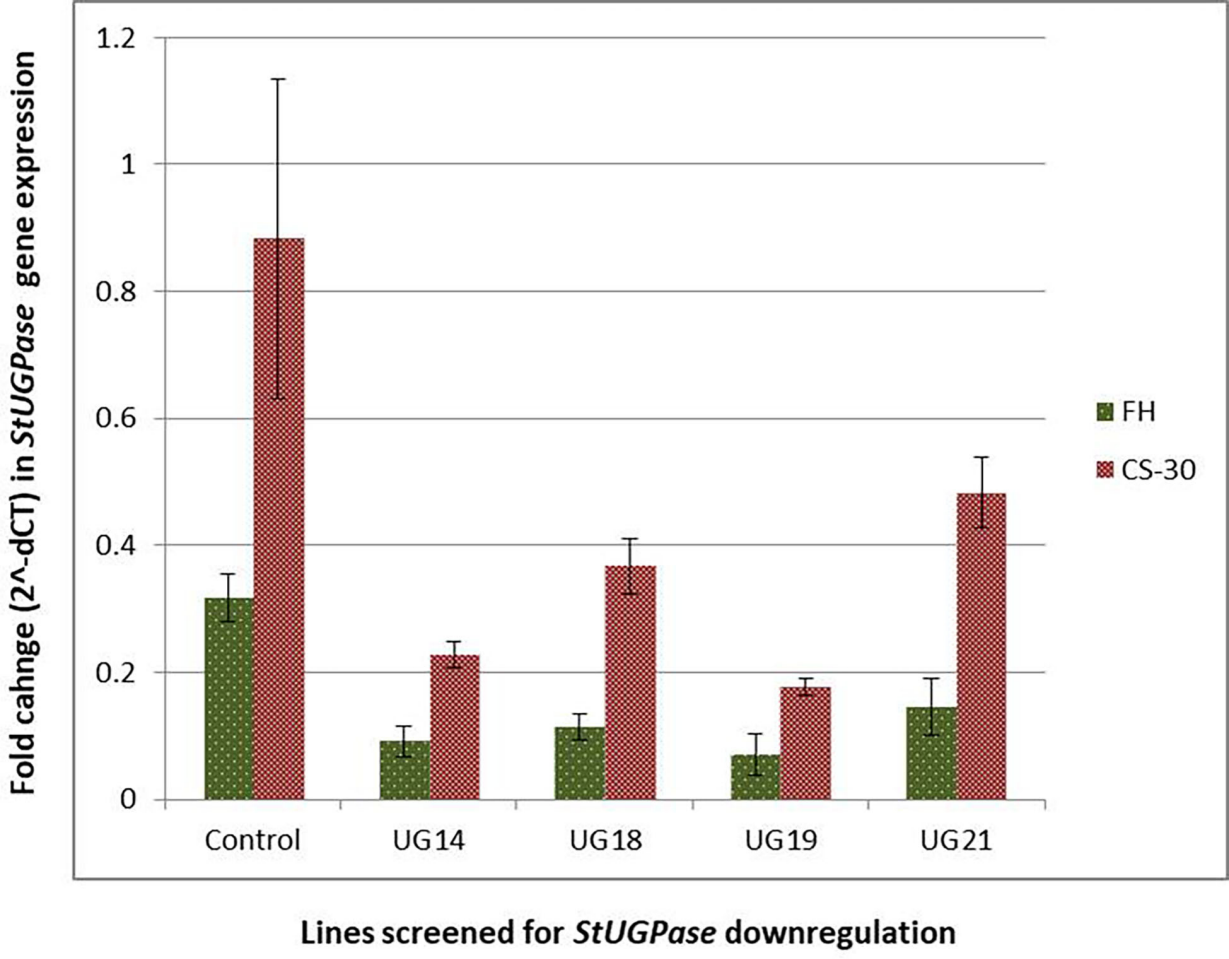
Reduction in *StUGPase* expression in the four RNAi potato transgenic lines of KC4 as revealed by Real-Time PCR analysis. Control, KC4C plant; UG14, UG18, UG19, and UG21 represents RNAi transgenic lines. FH, fresh harvest; CS-30, cold stored for 30 days.

Real-time PCR analysis was conducted to detect the extent of downregulation of the target gene, *StUGPase*, expression. cDNA was prepared from RNA isolated from both the freshly harvested and cold stored tubers of the four *UGPase* RNAi transgenic lines and wild-type control and used for real-time analysis. Many-fold reduction in the steady-state *StUGPase* transcript abundance was recorded in cold-stored tubers of the RNAi potato line was detected (**Figure 8**). The highest reduction (4.5 fold at FH and 5 fold at CS-30) in transcript level was observed in UG19. The level of reduction in *StUGPase* transcript in UG14, UG18, and UG21 was 3.5, 2.8, and 2.2 folds, respectively, at fresh harvest (FH) and 3.9, 2.4, and 1.8 folds, respectively, after cold storage for 30 days (CS-30) compared to that of the control. In comparison to the freshly harvested and reconditioned tubers, the level of *UGPase* expression was 2.51- 3.32 times higher in the cold stored tuber. But still, in both the conditions, the same pattern of downregulation of *UGPase* expression was observed.

## 4 Discussion

The data presented in the current study demonstrate that RNAi-mediated silencing of the *StUGPase* gene is an effective approach of reducing RS accumulation and consequently improving cold chipping attributes in processing potato cultivars. Based on previous studies the two enzymes, namely vaINV and UGPase, have been considered to be the two key regulatory enzymes of CIS. To date, considerable efforts were made to reduce vaINV activity and RS accumulation in potato tubers utilizing a multitude of molecular strategies, including overexpression of invertase inhibitors (Greiner et al., 1999; McKenzie et al., 2013) and silencing of the gene encoding vaINV (Zhang et al., 2008; Pattanayak et al., 2009; Bhaskar et al., 2010; Wiberley-Bradford et al., 2014; Zhu et al., 2014; Clasen et al., 2016; Yasmeen et al., 2022). Since vaINV is associated with the breakdown of sucrose into RS during CIS, the inhibition of vaINV activity could lead to the accumulation of sucrose in the tuber. Although sucrose does not influence potato chip colour, but it imparts an undesirable sweet taste in chips prepared from cold stored vaINV silenced potato (Pattanayak et al., 2009). This sucrose accumulation could further be prevented by manipulating another key enzyme, UGPase, since it has significant control over the flux of carbon toward sucrose biosynthesis and found to play a crucial role during CIS (Zrenner et al., 1993; Spychalla et al., 1994; Hill et al., 1996; Bagnaresi et al., 2008; Chen et al., 2008). Therefore, the present study was designed to suppress *StUGPase* expression by hpRNA-mediated silencing of *StUGPase* and consequent amelioration of CIS in potato. As UGPase is involved in many important biological functions it was perceived that complete knockout of the gene might have detrimental effects in growth and development. Hence, RNAi-mediated silencing of *StUGPase* was adopted considering the ease of hpRNA construct development and high efficacy of the technique (He and Hannon, 2004; Duda et al., 2014; Liang et al., 2015; Swiech et al., 2015). Previous attempts of silencing of *StUGPase* employing antisense approach demonstrated variable success. Zrenner et al. (1993) could not find any significant change in RS content despite 96% decrease in UGPase activity of antisense line, Spychalla et al. (1994) observed a significant change in the sucrose content of cold stored transgenic potato tubers expressing an antisense construct of *StUGPase*. In the present study, significant reduction of soluble sugar content (both sucrose and RS content) was observed in the selected four RNAi transgenic lines’ tubers of KC4 after cold storage for 30 days. Reduction of RS content was reflected in the colour of the chips prepared from cold stored transgenic tubers. Chip colour of the cold stored RNAi transgenic tubers was in the acceptable range (score of 2.5 to 3.0) compared to very dark brown colour (score of 6.5) in the cold stored control tubers. It was reported that RS level in cold stored potato should not exceed 160mg/100g tissue fresh weight to get acceptable colour in chips with score of 1-3 (Ezekiel et al., 2003). As both the RS content and chip colour score of cold stored RNAi transgenic tubers were at the border line of acceptance processing attributes of the selected RNAi transgenic potato lines were tested after reconditioning of tubers at room temperature (20-24° C) for 21 days. Room temperature reconditioning of cold stored potatoes is normally followed by the processing industries for improvement in colour and texture in the processed potato products like chips and fries (Ezekiel et al., 2003; Marwaha et al., 2010). In the present study, much improvement in sucrose and RS content and consequently in chip colour were recorded after reconditioning. Sucrose content in all the four RNAi lines was almost to that of the respective fresh harvest level, and RS content was reduced to half of the respective level after cold storage and also almost half compared to that of control after reconditioning. That at each point of the study (fresh harvest, 30 days of cold storage and after reconditioning) soluble sugar level (both sucrose and RS content) was almost 50% less in the RNAi lines compared to that of control indicates the efficacy of the strategy.

Downregulation of the target gene was recorded in all the selected transgenic lines (54-72 % in FH and 45-80% under CS-30 conditions). *StUGPase* expression was reported to be substantially up-regulated in potato tubers at low temperature (Spychalla et al., 1994; Borovkov et al., 1996) which is also evident in the current study. *StUGPase* expression was enhanced many folds in untransformed WT plants under cold storage. Despite that, the extent of *StUGPase* down regulation was almost similar during both at fresh harvest and after cold storage. Detection of *StUGPase*-specific siRNAs in cold stored RNAi transgenic tubers, but not in cold stored control tubers, confirms that this down regulation of *StUGPase* expression was mediated by these siRNA mediated degradation of *StUGPase* mRNA. This indicates that RNAi-mediated silencing was not inhibited in cold, and was effectively operational during cold storage (Szittya et al., 2003). Although we could not find a strict correlation among the extent of down regulation of *StUGPase* expression, reduction of soluble sugar content in the cold stored RNAi transgenic tubers, and chip colour. This could be due to the influence of some other factors on tuber sugar content and chip colour. It is important to note that the tuber sugar content and chip colour are influenced by many factors like disease stress, temperature and water stress, management practices, and mechanical handling (Kumar et al., 2004).

## 5 Conclusion

Amelioration of CIS in transgenic potato developed by RNAi-mediated silencing of *StUGPase* demonstrates the important role played by the enzyme in the regulation of sucrose biosynthesis and the accumulation of RS in the cold-stored potato tubers. A considerable improvement in the cold-chipping ability of UGPase RNAi transgenic potato in terms of reduction in sucrose and RS content and improvement in chip colour demonstrates the efficacy of the strategy. No growth and developmental abnormalities were observed during the net house trial. The apparent variation in yield was due to the planting of different size baby seed tubers. However, the selected four lines showing a 2 to 5 fold reduction in *StUGPase* expression had a comparable average yield to that of the control. Considering the vital role UGPase plays in plant growth, development, survival under stress conditions, and also in CIS it can be said that our strategy could strike a balance between induction of a moderate level of silencing of this essential gene and achieving considerable improvement in reduction of soluble sugar content and improvement in chip colour. Our approach, being less disruptive, might be the choice to get commercial success in near future. In the future, modern genome editing tools can also be employed to get any mutant with optimum silencing of *StUGPase* and development of transgene free CIS tolerant potato lines.

## Data availability statement

The original contributions presented in the study are included in the article/**Supplementary Material**, further inquiries can be directed to the corresponding author/s.

## Ethics statement

This article does not contain any studies involving animals or human participants as objects of research.

## Author contributions

Conceiving and designing the experiment were done by DP. Data curation and analysis were done by SJ, JT, and SB. The experiment was conducted by SJ, KR, ST, MS, and KP. Supervision of the research work by DP. Writing of the original draft by SJ. Review and editing of the manuscript by DP, KR, KM, JV, and RS. All authors contributed to the article and approved the submitted version.
