# Supplementary material for: Amelioration of cold-induced sweetening in potato by RNAi mediated silencing of StUGPase encoding UDP-glucose pyrophosphorylase
Source: Front Plant Sci. 2023 Feb 17;14:1133029. doi: 10.3389/fpls.2023.1133029 (PMC9981964; doi:10.3389/fpls.2023.1133029)

**Supplementary Table 1.** Primer details of *StUGPase* gene and GBSS intron used for PCR amplification

| **SN.** | **Primer name** | **Sequence (5’-3’)** |
| --- | --- | --- |
| 1. | **UGPase sense F** | ctagta***tctaga***gtcccaaat cag ttattgaagttc  (*Xba*I is in italics bold; Tm for targeted region 52.5° C) |
| 2. | **UGPase sense R** | ctactc***gag ctcggtaccagatctccatgggtcgac ccc ggg***atatga gat taaggtgccacc  (*Sac*I, *Kpn*I, *Bgl*II, *Nco*I, *Sal*I &*Xma*I sequentially are in italics bold; Tm for targeted region 52.1° C) |
| 3. | **UGPase antisense F** | ctactc***gag ctc***gtcccaaat cag ttattgaagttc  (*Sac*I is in italics bold; Tm for targeted region 52.5° C) |
| 4. | **UGPase antisense R** | ctacgc***ggatcc***atatga gat taaggtgccacc  (*Bam*H1 is in italics bold; Tm for targeted region 52.1° C) |
| 5. | **GBSS intron F** | cttacc***ccc ggg***aactctcctggaagg tag gtg  (*Xma*I is in italics bold; Tm for targeted region 55.8° C) |
| 6. | **GBSS intron R** | ctactc***gag ctcggtaccagatctccatggggatcc***aggttcctg cat gaacac cag  (*Sac*I, *Kpn*I, *Bgl*II, *Nco*I &*Bam*HI, sequentially, are in italics bold; Tm for targeted region 57.6° C) |

**Supplementary Table 2.** Primers sequence used for PCR screening of putative transgenic lines

| **S.No** | **Primer name** | **Sequence (5’-3’)** |
| --- | --- | --- |
| **1.** | **UGPaseS F** | **CTAGTA*TCTAGA*GTCCCAAATCAGTTATTGAAGTTC** |
| **2.** | **GBSS In R** | **CTACTC*GAGCTCGGTACCAGATCTCCA TGGGGATCC*AGGTTCCTGCATGAACACCAG** |

**Supplementary Table 3.** Components of the PCR reaction mixture along with their volumes (µL)

| **S.No.** | **Components** | **Volume(for 25 µl)** |
| --- | --- | --- |
| 1. | DNA | 0.5 |
| 2. | Taq  Buffer A | 2.5 |
| 3. | 2.5 mM dNTP’S | 2.0 |
| 4. | Forward Primer | 0.5 |
| 5. | Reverse Primer | 0.5 |
| 6. | Nuclease free water | 18.7 |
| 7. | Taq Polymerase Enzyme | 0.3 |
| 8. | Total Reaction mixture | 25.0 |

**Supplementary Table 4.** Primers sequence used for qRT- PCR screening of selected transgenic lines

| **SN.** | **Primer** | **Primer sequence (5’-3’)** | |
| --- | --- | --- | --- |
| **1.** | **UGPaseqRT** | **Forward** | **AGATAACTTGGGTGCCATTGTTG** |
|  |  | **Reverse** | **GGTGCCACCTTTGACATCAGCT** |
| **2.** | ***StEF1α*** | **Forward** | **ATTGGAAACGGATATGCTCCA** |
|  |  | **Reverse** | **TCCTTACCTGAACGCCTGTCA** |

**Supplementary Figure 1.** Nucleotide sequence of *S. tuberosum* UGPase cDNA sequence (GeneBank Sequence ID: [D00667.1](https://www.ncbi.nlm.nih.gov/nucleotide/D00667.1?report=genbank&log$=nuclalign&blast_rank=3&RID=Z2NH45W9014))

1 gaattcggca cgaggtttat ttaaagagac aaattttaca ttcacacact atatctatca

61 ctcttctctc catactctct gctcctcgag aactttctct tctcatttct ctctgtagat

121 cacaatcttc ttcttcgcta tggctactgc tactactctt tctcctgctg atgccgaaaa

181 gctcaacaat ctcaaatctg ccgtcgccgg tcttaatcaa atcagtgaaa atgagaaatc

241 tggatttatt aaccttgtcg gtcgctatct aagtggagaa gcgcaacaca ttgactggag

301 taagatccag acgccaactg atgaggtggt ggtgccatat gacaagttag cacctctttc

361 tgaagatccc gcagagacta agaagctttt ggacaaactt gttgtcctga agctcaatgg

421 aggcttggga acaacaatgg gatgcactgg tcccaaatca gttattgaag ttcgtaatgg

481 tttgacattc cttgacttga ttgtcaagca aattgaggcc ctcaatgcca aattcggatg

541 cagtgttccc ctgcttttga tgaattcgtt caacacccat gatgatacac tgaagattgt

601 agaaaaatat gcgaactcaa acattgatat tcatacattc aatcagagcc agtaccctcg

661 cctggttacg gaagactttg ccccacttcc atgcaaaggc aattctggaa aagatggatg

721 gtaccctcca ggtcatggtg atgttttccc ttctttgatg aatagtggaa agcttgatgc

781 actactagca aagggaaagg aatatgtctt tgttgcaaac tcagataact tgggtgccat

841 tgttgatttg aaaatcctaa atcatctgat cctaaacaaa aatgagtatt gcatggaggt

901 tactcccaaa actttagctg atgtcaaagg tggcacctta atctcatatg aaggaaaagt

961 acagctgctg gaaatagcac aagtccctga tgaacatgtc aatgaattca agtcaattga

1021 aaaattcaaa attttcaata ccaacaactt gtgggtgaat ctgagtgcta ttaaaagact

1081 tgtagaagca gatgcactca agatggagat tattcccaac ccaaaggaag tggacggagt

1141 caaagttctt caacttgaaa ctgctgccgg tgctgcaatt aagttttttg accgggcaat

1201 tggtgctaat gttcctcgat ctcgtttcct tcccgtgaaa gcaacttcag atttgctcct

1261 tgttcagtct gatctttaca ccttgactga tgagggctat gttatccgaa acccggccag

1321 gtcgaatcca tccaacccat ccatcgagtt gggacctgaa ttcaagaagg tggccaactt

1381 cttaggccgt ttcaagtcta ttcccagcat cattgatcta gatagcttga aggtgaccgg

1441 tgatgtatgg ttcggatccg gcgttaccct aaaggggaaa gtgactgttg cagccaaatc

1501 aggagtgaag ctagaaattc cagatggtgc tgtgattgca aacaaggata tcaatggacc

1561 tgaggatata tagagtagct gctggtgcca attcgacgtc gcggagtgtg aagcaagtat

1621 gtttgtagtg aatgtaaaat ttgcttcttt aaataaacaa acagttttgc ttgttttgat

1681 ttaatggaac cattgataca atacaaatga aatgactata tcagagtaac ttgttcctta

1741 aaaactcgtg ccgaattc

**Supplementary Figure 2**. Nucleotide sequence region (450-949) selected for the development of inverted repeat gene construct for PTGS of UGPase gene.

421 g tcccaaatca gttattgaag ttcgtaatgg

481 tttgacattc cttgacttga ttgtcaagca aattgaggcc ctcaatgcca aattcggatg

541 cagtgttccc ctgcttttga tgaattcgtt caacacccat gatgatacac tgaagattgt

601 agaaaaatat gcgaactcaa acattgatat tcatacattc aatcagagcc agtaccctcg

661 cctggttacg gaagactttg ccccacttcc atgcaaaggc aattctggaa aagatggatg

721 gtaccctcca ggtcatggtg atgttttccc ttctttgatg aatagtggaa agcttgatgc

781 actactagca aagggaaagg aatatgtctt tgttgcaaac tcagataact tgggtgccat

841 tgttgatttg aaaatcctaa atcatctgat cctaaacaaa aatgagtatt gcatggaggt

901 tactcccaaa actttagctg atgtcaaagg tggcacctta atctcatat

**Supplementary Figure 3.** Nucleotide sequence corresponding to 3821-3925 nt of potato GBSS genomic DNA sequence (GenBank Acc. No. X58453; Potato gene for granule-bound starch synthase).

3781 aactctcctg gaaggtaggt

3841 gtcaaattga taatttgcgt aggtacttca gtttgttgtt ctcgtcagta ctgatggatg

3901 ccaactggtg ttcatgcag

**Supplementary Table 5**. Yield data of 22 lines grown in the transgenic net house.

| **S.No.** | **Line No.** | **Average Yield (gram per plant)** |
| --- | --- | --- |
| 1. | KC4 | 305.06±26.62 |
| 2. | UG2 | 149.16±24.57 |
| 3. | UG4 | 327.38±37.74 |
| 4. | UG5 | 249±26.87 |
| 5. | UG6 | 108.42±21.75 |
| 6. | UG7 | 250.26±32.52 |
| 7. | UG8 | 193.12±31.31 |
| 8. | UG10 | 243±34.29 |
| 9 | UG11 | 91.6±10.88 |
| 10 | UG12 | 269.8±11.09 |
| 11. | UG13 | 160.88±23.58 |
| 12. | UG14 | 300.46±23.82 |
| 13. | UG16 | 188.33±41.69 |
| 14. | UG 18 | 231.86±22.63 |
| 15. | UG 19 | 287.93±17.65 |
| 16. | UG 21 | 270.13±18.11 |
| 17. | UG24 | 132.33±15.67 |
| 18. | UG26 | 140.8±20.08 |
| 19. | UG27 | 116.8±32.43 |
| 20. | UG31 | 147.1±16.70 |
| 21. | UG35 | 198.8±44.30 |
| 22. | UG36 | 101±26.64 |

**Supplementary Table 6.** Sugar content and chips colour scores of selected transgenic lines at various evaluation stages (Fresh Harvest, Cold stored and reconditioned tubers)

| **Lines** | **Glc content** | **Fru content** | **Suc content** | **Total RS** | **Total soluble sugar** | **Hexose: Sucrose ratio** | **Chips colour** |
| --- | --- | --- | --- | --- | --- | --- | --- |
| **Fresh harvest** | | | | | | | |
| KC4 C | 8.36±0.06 | 3.65±0.06 | 320.16±13.20 | 12.03±0.0 | 332.18±13.20 | 0.04 | 1 |
| UG14 | 5.019±0.13 | 3.10±0.03 | 167.90±5.32 | 8.13±0.16 | 176.03±5.16 | 0.05 | 1 |
| UG18 | 3.28±0.08 | 1.60±0.03 | 180.70±14.06 | 4.89±0.10 | 185.59±14.087 | 0.03 | 1 |
| UG19 | 4.37±0.09 | 3.12±0.20 | 140.88±4.31 | 7.50±0.20 | 148.39±4.37 | 0.05 | 1 |
| UG21 | 4.046±0.18 | 1.95±0.08 | 170.39±7.46 | 6.00±0.26 | 176.39±7.55 | 0.035 | 1 |
| **Cold stored tuber at 4°C for 30 days** | | | | | | | |
| KC4 C | 153.36±5.85 | 130.25±4.7 | 1313.83±15.6 | 283.62±10.55 | 1597.45±10.08 | 0.21 | 6.5 |
| UG14 | 62.03±2.23 | 64.14±1.58 | 789.72±19.56 | 126.18±3.79 | 915.90±18.71 | 0.16 | 2.5 |
| UG18 | 55.03±2.24 | 74.43±1.75 | 909.85±13.71 | 129.46±2.49 | 1039.32±12.09 | 0.14 | 3.0 |
| UG19 | 65.37±2.52 | 64.81±2.33 | 812.38±15.39 | 130.20±3.82 | 942.57±11.62 | 0.16 | 2.5 |
| UG21 | 58.34±5.46 | 62.11±8.59 | 707.78±19.16 | 162.97±5.99 | 828.24±24.81 | 0.17 | 3.0 |
| **Reconditioned tubers at room temperature for 30 days** | | | | | | | |
| KC4 C | 70.24±0.78 | 40.36±0.79 | 227.93±5.67 | 110.61±1.55 | 338.54±4.89 | 0.48 | 3.5 |
| UG14 | 35.62±0.84 | 39.28±1.73 | 189.94±2.56 | 74.91±1.61 | 264.85±3.43 | 0.39 | 1.5 |
| UG18 | 33.14±0.21 | 32.07±0.68 | 188.94±4.84 | 65.22±0.74 | 254.16±4.68 | 0.34 | 2.0 |
| UG19 | 26.41±2.34 | 22.32±5.14 | 176.87±5.31 | 48.74±2.84 | 225.61±2.72 | 0.27 | 1.5 |
| UG21 | 25.55±0.88 | 22.70±0.09 | 181.28±3.18 | 48.26±0.78 | 229.54±2.49 | 0.26 | 2 |

**Supplementary Figure 4.** PCR screening of *UGPase* RNAi putative transformants of potato cultivar, KC4, using *StUGPase*-S F and GBSS-Int R primers. Lanes: 100bp, 100 bp DNA marker; +ve, positive control; WC, water control; WT, wild type sample; UG2-UG35, *UGPase* RNAi transgenic potato lines screened. Bands indicate amplification of̴ 600 bp fragments corresponding to *StUGPase*-S and *StGBSS*-Int region of the hpU transgene.


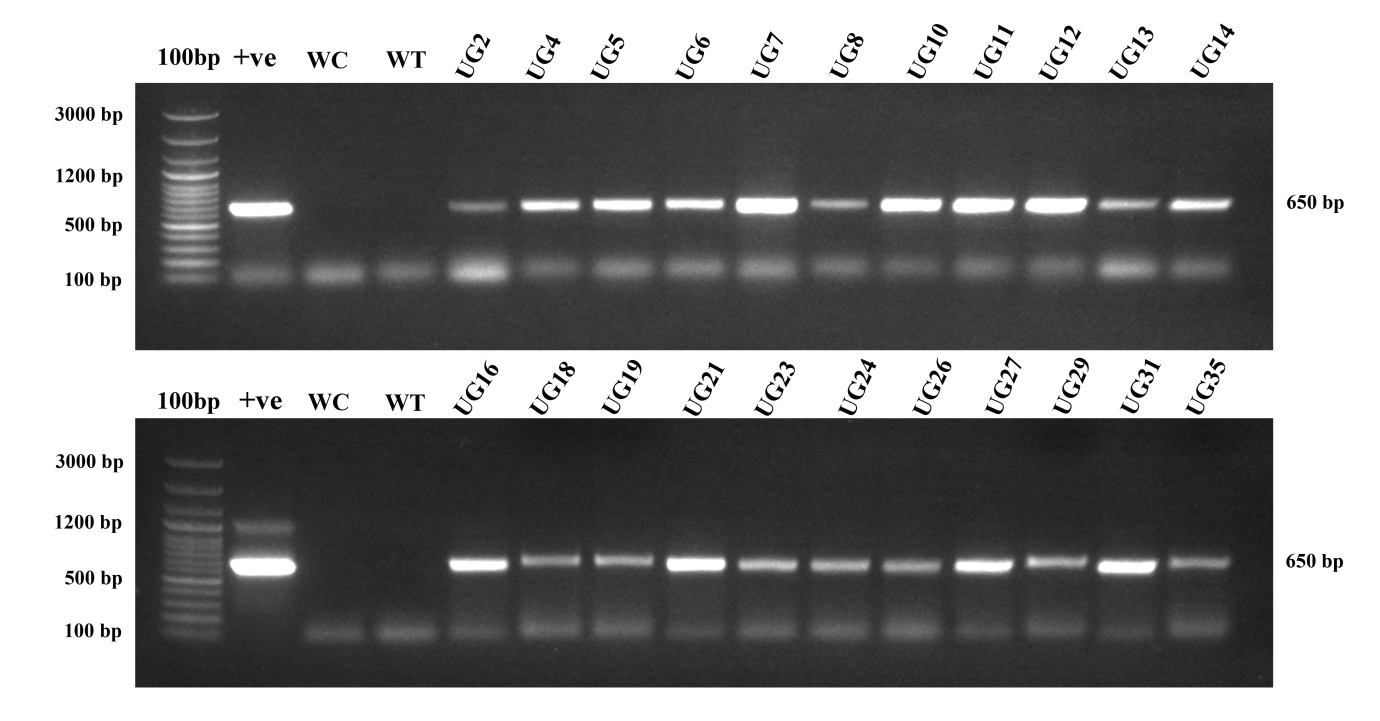


**Supplementary Figure 5.** RT-PCR screening of *UGPase* RNAi putative transformants of potato cultivar, KC4, using *StUGPase*-S F, and GBSS-Int R primers. Lanes: 1kb, 1 kb DNA marker; WT, wild type sample; -ve, negative control; WT, wild type sample; UG2-UG35, *UGPase* RNAi transgenic potato lines screened.Bands indicate amplification of ̴ 600 bp fragments corresponding to *StUGPase*-S and *StGBSS*-Int region of cDNA derived from transcripts of the hpU transgene.


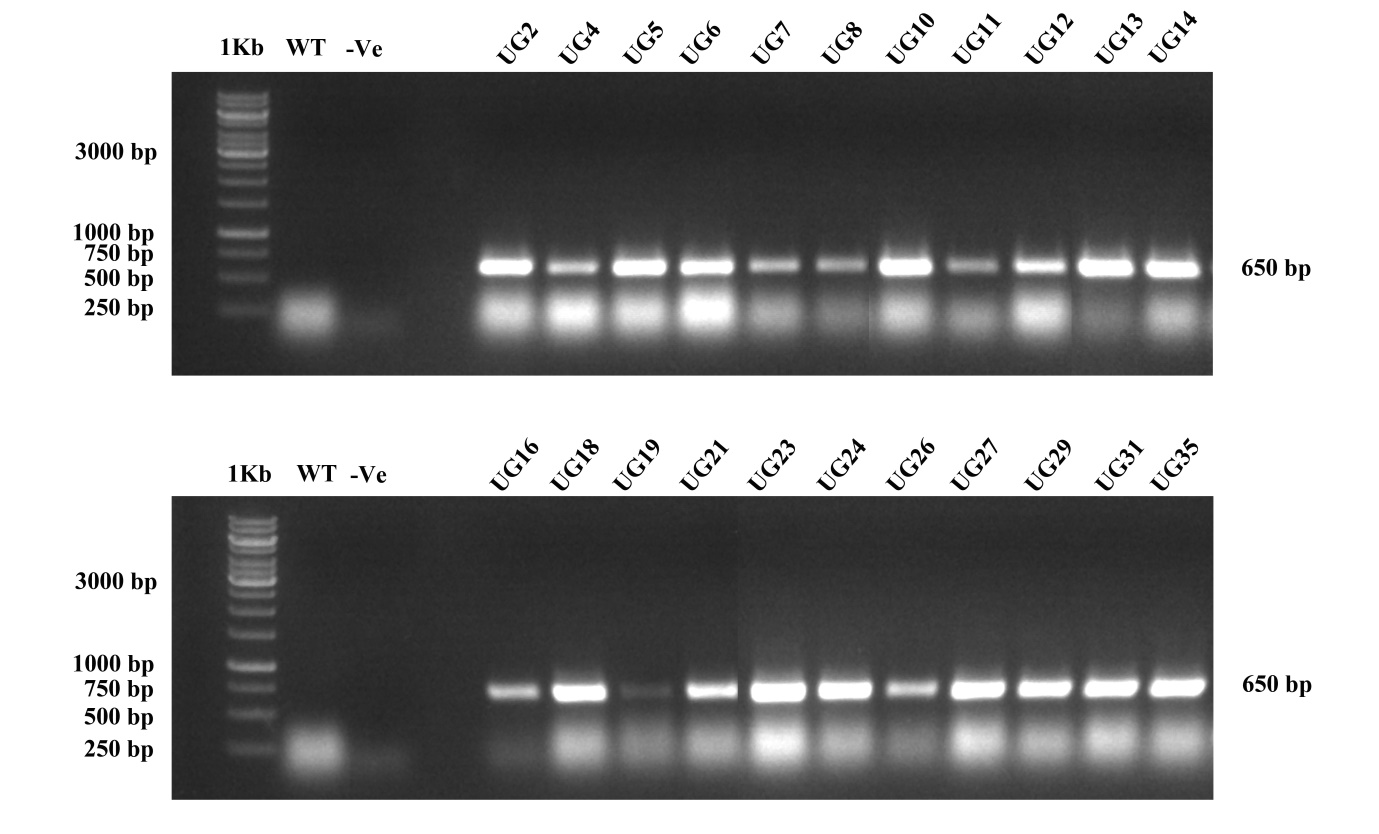

Supplement: Supplementary file 1 [file DataSheet_1.docx]
