# Supplementary figures and images for: Amelioration of cold-induced sweetening in potato by RNAi mediated silencing of StUGPase encoding UDP-glucose pyrophosphorylase
Source: Front Plant Sci. 2023 Feb 17;14:1133029. doi: 10.3389/fpls.2023.1133029 (PMC9981964; doi:10.3389/fpls.2023.1133029)

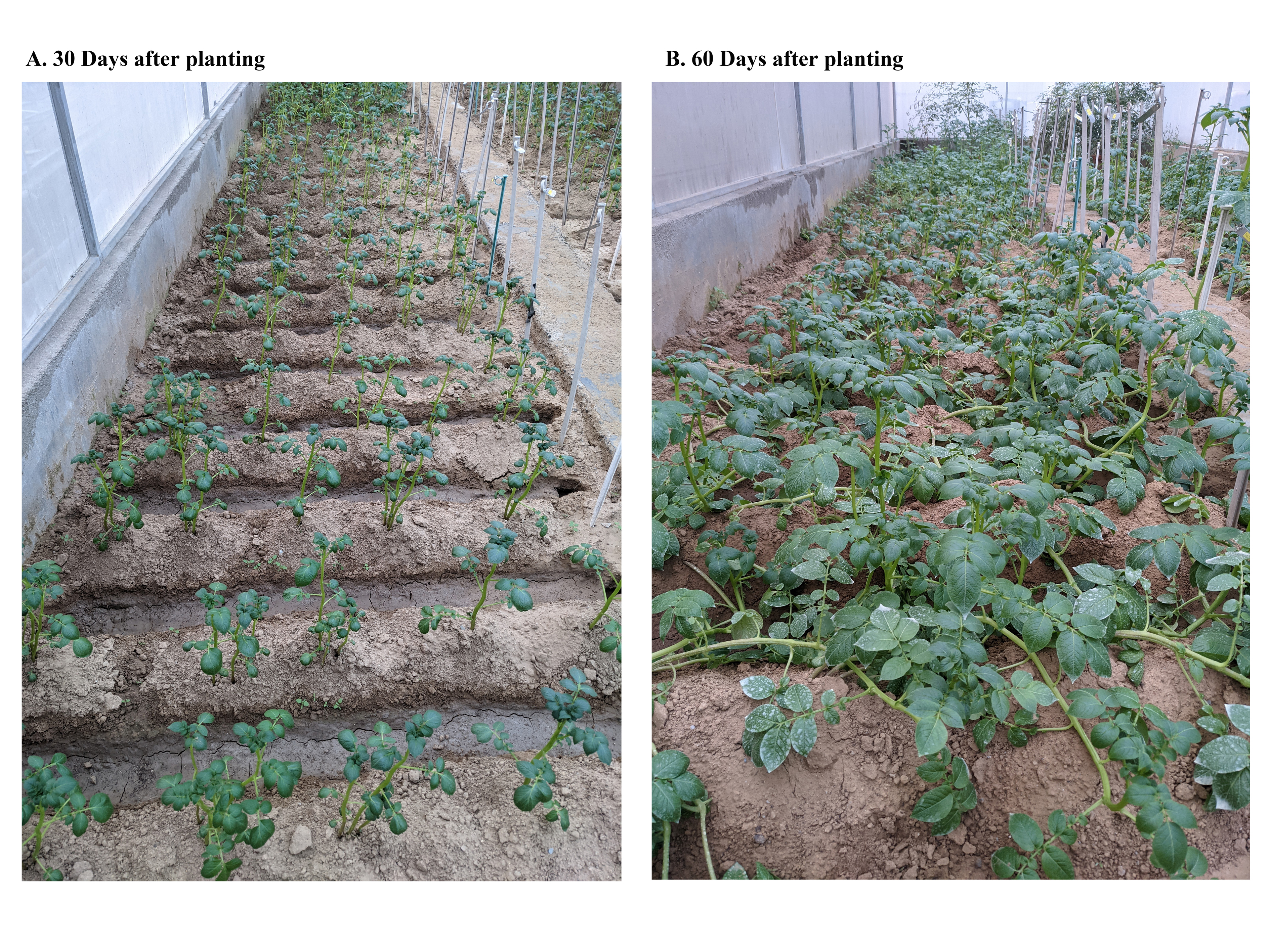

Supplement: Supplementary file 2 [file Image_1.jpg]
